# Functions of digital learning within the international mobility programme – perspectives of university students and staff from Europe

**DOI:** 10.1007/s10639-021-10855-y

**Published:** 2021-12-30

**Authors:** Joanna Leek, Marcin Rojek

**Affiliations:** University of Lodz, Lodz, Poland

**Keywords:** Functions of learning, International mobility, Digital learning, Higher Education in Europe

## Abstract

This paper is based on research studies conducted in the academic community of students and staff members (teachers, researchers and administrative staff) from 16 European universities that focus on digital learning in international mobility. The context of our qualitative study is digital learning during an international mobility scheme when university staff and students do not go abroad for their mobility programme but take courses offered by a partner university from home. By taking the perspectives of both of these academic groups, we aimed to arrive at a clearer understanding of how the digital environment supports digital learning within mobility, ascertain the functions of digital learning and describe the opportunities and challenges that are presented to students engaged in international mobility. Empirical data was gathered using questionnaires and focus group interviews. This study puts forward the assertion that distinctive features of learning in a digital environment within international mobility are systems thinking, self-directed learning and focus on course content. Digital learning environments support motivation to learn, and independence in gaining knowledge. In international digital learning, the online courses of which are characterized by their innovative pedagogical and assessment practices, students and staff become more autonomous in their learning, and more willing to open up to meeting the challenges encountered in various educational settings. Digital learning in the context of mobility means giving meaning to one’s own activity in a digital environment and extension of the course content, meaning oral expression such as discussing and interacting with teachers and peers.

## Introduction

Learning spaces in University classrooms are equipped with more than chairs and tables. Computers or the Internet are nowadays commonly used in learning, and create new symbolic learning conditions. Fairly new environment of learning with digital tools is being created by international mobility exchange, and the opportunity for students to work or study abroad whilst undertaking their degree programme, or for University staff to gain knowledge or skills during internships or training sessions.

The purpose of this study is to understand and to explain University students’ and staff’ digital learning within an international mobility programme, like Erasmus + programme in the case of Europe, where mobility participants do not go abroad to carry out their mobility activity abroad (unlike the physical mobility case) but nevertheless learn within their mobility throughout online courses (taken from home, without travelling), led by teachers from other countries, accompanied by simultaneous interactions within online activities. Empirical data was gathered using questionnaires and focus group interviews. To scope experiences with digital learning in the environment of international mobility of 160 students and 103 staff from 12 universities, located in Germany, Portugal, France, Brussels, Poland, Hungary. The background for comparisons were experiences with traditional mobility where students or staff go abroad to carry out mobility activities in a partner university. Incorporating experiences with learning into traditional mobility aimed at gaining a wider range of experiences with different mobility programmes, firstly, to find out the distinctive features of learning within mobility and then to detect those features that relate to learning in the digital environment during academic mobility. In adopting a comparative approach to studying academic staff and students’ experiences, we followed the advice of Noesgaard and Ørngreen (2015) who claim that the effectiveness of learning in a digital environment can be approached in different ways, depending on the aim of the e-learning. In the questionnaire study, international mobility of students and staff from Europe was used to gather information on experiences with mobility in general (vertical differentiation of learning background – learning experiences gained at another university during mobility) and in focus group interviews, to gain information on learning in an digital environment (horizontal differentiation of learning background – digital tools as one of the opportunities available to gather learning experiences in academia). What combines these two differentiations, are the experiences that students and staff members had while participating in learning in an international environment, experiences with digital tools during their mobility, cooperation that existed during mobility, the flexible way that work was structured to create interactive and engaging content, all of which are distinctive features of the digital learning environment. (Siemens, 2014).

With this study, we claim that distinctive features of learning in a digital environment within international mobility are systems thinking, self-directed learning and focus on course content. Digital learning within mobility shapes students’ career identity, and is perceived by students as an opportunity to find themselves in advantaged positions in the labour market. In relation to staff, digital learning has professional development value. The digital learning environment is something new for adult mobility participants, and generally speaking, they do not usually associate it with their school learning, which is sometimes viewed negatively. Therefore adults do not have prejudices against this type of learning – something that even gives them partial satisfaction. One of the advantages of digital learning in relation to international mobility is that academic staff can with this kind of learning focus on performing tasks and practicing rather than theory, which is a characteristic feature of adult learning. Respondents mostly cited gaining digital skills, professional knowledge and skills development as the main benefits of this kind of education. The research results allow us to conclude that digital learning differs from traditional (physical) learning. Digital learning within international mobility is a very particular way of experiencing the world, therefore it should be implemented in educational practice parallel to traditional learning, and not presented as alternatives.

## Internationalization through mobility

The study is underpinned by the theory on adult development, particularly by Kegan’s cognitive-developmental theory (Kegan, 1982, 1998) that describes the different developmentally-related ways in which adults can view their world and problems experienced in it. In particular, Kegan emphasizes that as people grow, they are moving objects, and key ideas in their purview from “subject to object”. In other words, they can take these notions and move from being subject to them to holding them as object. This adult development perspective suggests that learning courses within mobility have potential to provide international learning experiences that help students to view their own systems with greater distance and perspective. As yet, however, there is no empirical account of the extent to which this kind of learning is being assessed in relation to their own expectations. This gap is being filled with our study.

Internationalization is viewed as a process acting within HE institutions to integrate elements of internationalization into areas of research and teaching, but also includes student services. This term can be also considered in terms of academic mobility, and part of institutional culture (Tanhueco-Nepomuceno, 2019) that is a solid component of the universities offering opportunities and challenges (Knight, 2015). Altbach and Knight (2007) perceive internationalization as part of an academic system, the institutions and individuals that form its policies and programmes or foreign elements in local programmes (Tran, 2018; Beelen & Jones, 2015). Another fairly common form of internationalization is student exchange, also referred to as international mobility. Studies on mobility emphasize the impact that students have on intercultural understanding (Messelink, Maele & Spencer-Oatey, 2015), sense of self-identity and adaptive capacities (Jacobone & Moro, 2015). Learning outcomes of mobility are “curiosity, initiative, risk-taking, suspension of judgment, cognitive flexibility, tolerance of ambiguity, cultural humility and resourcefulness that lie at the heart of international learning” (Bennet, Maton & Kervin, 2008, p. 20).

Mobility is also connected with the pressure put on universities to equip graduates with skills to work in an international environment in response to the demand for employable university graduates (Leask, 2013). In the European context, mobility of HEI is associated with the Erasmus programme, which is perceived as “a strategic platform for the promotion of human development” (Martínez-Usarraldea, Pausá & García-López, 2017, p. 107), establishing joint international research programmes and degrees. Mobility increases opportunities for educational experiences and international learning, therefore it is particularly an instrument for building the potential of individuals and societies. It enables knowledge to flow between countries and strengthen international research collaboration, with potential benefits at both individual, institutional and national levels. Moreover, the possibility of international learning reduces the likelihood of local obstacles such as poverty, social exclusion, digital exclusion, and discrimination (Karady, 2002). Recent studies suggest that student mobility enables young women to flee countries where they are denied educational opportunities because of gender-related norms such as family expectations of early marriage (Martin, 2017).

## Research questions

For the study, we formulated following research questions:

1. What are features of students’ and staff learning within international mobility programmes?
2. What are functions of digital learning within international mobility programmes?
3. What is the role of mobility in the learning practices?

These questions were formulated after an in-depth analysis of the literature containing the results of previous research on the educational dimension of students’ learning, virtual learning and international mobility (Tanhueco, Tanhueco-Nepomuceno, 2019; Cairns, 2018; Tran,, 2018; Martínez-Usarraldea, Pausá & García-López, 2017; Knight, 2015; Martin, 2017; Beelen & Jones, 2015; Messelink, Maele, Spencer-Oatey, 2015; Jacobone & Moro, 2015; Leask, 2013; Bennet, Maton & Kervin, 2008; Altbach & Knight, 2007; Karady, 2002) and finding that there is insufficient explanation for students learning in the virtual world through international mobility or foregoing explanations were built on the research carried out among students only, without taking into account other important “actors” in higher education such as academic teachers and mobility programme coordinators.

## Method

**Chart 1:**
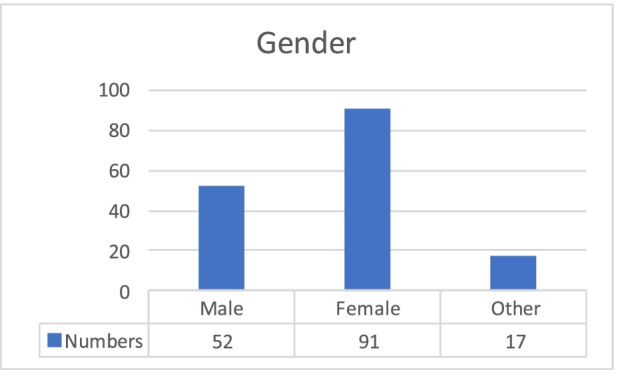
Gender of students. **Source:** original study

**Chart 2:**
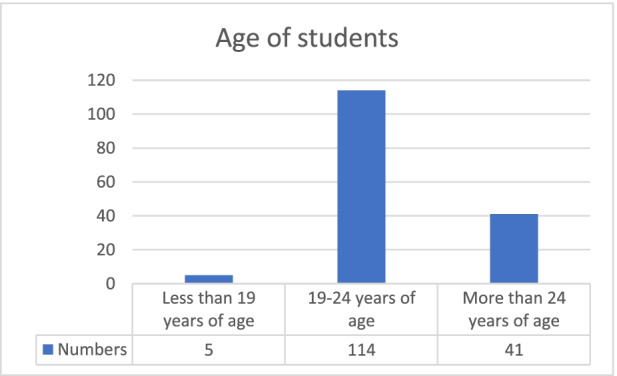
Age of students. **Source:** original study

**Chart 3:**
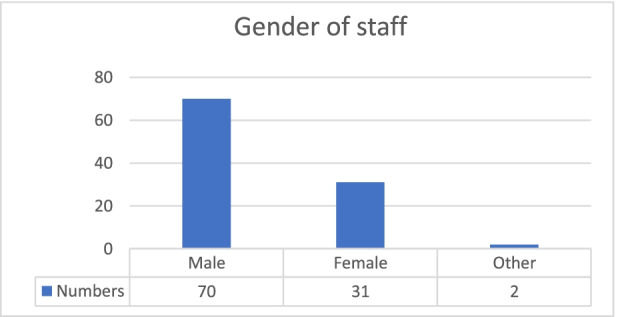
Gender of staff. **Source:** original study

**Chart 4:**
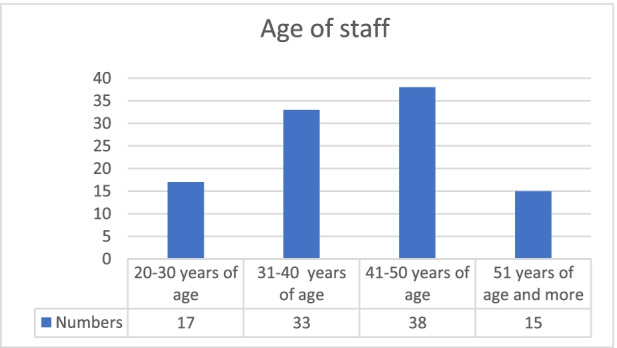
Age of staff. **Source:** original study

The study was conducted throughout the academic spring and autumn semesters of 2020, and was divided into two parts. First, empirical data was gathered from 160 students and 103 university employees (students, teachers, researchers and administrative staff from 12 universities that took part in international mobility in last 36 months), using an online questionnaire. The questionnaire study (in English) served a diagnostic purpose, aiming to identify both the experiences of students and staff employees and the causes, sources, circumstances and determinants of students’ and staff international learning. Participants were selected based on their experience with mobility. Selection criteria for students were participation in at least one mobility scheme in last 18 months, and for employees: teaching students from international mobility programmes (academic teachers/researchers) or students’ administrative service (administrative staff) (Charts 1, 2, 3 and 4).

This questionnaire was followed by 16 focus group interviews with 78 students from 6 universities, located in Germany, Portugal, France, Poland, Hungary. Interviews were held online using Zoom or MS Teams between October and December 2020. The approximate duration of interviews was between 45 to 70 min. Three to 6 students took part in each group interview. The focus group instructions (questions) were administrated by intermediates (usually 2 per interview) from partner institutions involved in the “…..” project, who were responsible for obtaining consents regarding the study participation, asking questions, and recording the interviews. These persons were defined as being ‘third party’ in our research process, in accordance with the term used by Howells (2006). Focus group interviews were aimed at finding out features and functions of digital learning within international mobility. At this stage of research, our aim was to find out learning experiences, especially with regard to learning functions in their life. However, our research approach was not a typical example of a mixed method (Brake, 2011; Creswell, 2013) as we did not carry out the two stages simultaneously, but one after the other.

## Research tools

For the questionnaire study, two questionnaires (one for students and one for academic staff) with closed and open-ended questions were developed. After initial analysis of questionnaire responses, we developed questions for focus group interviews concerning people’s personal experiences with international learning, things that influenced their learning practices and how personalized international learning changed students and their lives.

## Data analysis

The relationship between people and technology (e.g. Internet, ICT or digital tools) can be studied and explained in different ways. A process approach can be used (Beaudry & Pinsonneault, 2001) referring to the transactional theory of coping with stress (Lazarus & Folkman, 1984). The technology acceptance range is also used, for example Unified Theory of Acceptance and Use of Technology (UTAUT), Diffusion of Innovation Model (DOI) or Technology Acceptance Model (TAM). These ranges allows researchers to concentrate on the relationship between technology adoption and the variables that influence it. The ranges methods are important for developing contemporary knowledge about various aspects of the usage and implications of technology. However, they are characteristic for quantitative approach, and require mathematical procedures of analysis. Our study is qualitative, and model of data analysing which we adopted was distinctive and grounded in a qualitative approach.

**Chart 5:**
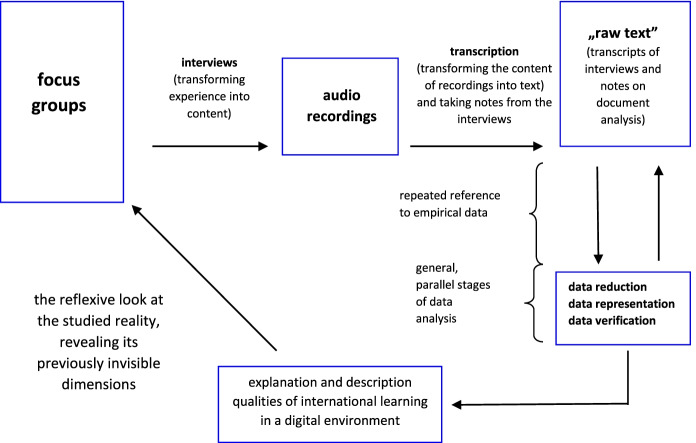
The process of collecting and analyzing the data. **Source:** original study

Analysis was carried out according to the Chart 5, which we developed based on Miles, Huberman & Saldaña (2014) concept of qualitative data analyzing.

After transcription of focus group interviews, the material collected both from questionnaires and focus group interviews was first read carefully, next, the main code segments such as “first thoughts about international learning in digital environment”, “experiences with international learning in digital environment”, “changes in learning practices”, “personal experiences”, “problems experienced during international learning in digital environment” and “main result from mobility with digital tools” were generated. Codes were the foundation for our main modes of thinking about and interpreting data (Gibbs, 2007, p. 79–82).

Then we identified links between different codes belonging to one category (intra-case analysis) and to different categories (cross-case analysis). Following this scheme, we aimed at obtaining a holistic (systemic, integrated) context, its logic and principles of international learning in a digital environment.

## Main findings – students

In the opinion of students, the best length of digital learning in international mobility is between 2 weeks and 2 months (40%). In second place in order of preference: 2–6 months (25%), up to 2 weeks (14%), longer than 12 months (3%), 6–12 months (1%), different length (17%). In an interview, students were asked to explain their choices, emphasizing that short-term mobility was associated with rapidly adapting to using digital tools and losing motivation if the online courses were too long. Examples of answers:

*For this type of learning, I think a few weeks would be enough or I would start to lose some motivation; I believe it is easier to adapt to something digital, but it would probably become exhausting after some time; It can become very long if you're always sitting in front of your PC; I guess that thanks to digital tools students might be able to adapt faster; Digital tools face the risk of losing their appeal quicker than actual visits.*

We asked students what knowledge, skills and attitudes are necessary to take part in international mobility. The responses were categorized as follows (italics font – examples of students voices):

- living in a foreign country
  *It helps if you know something about how to rent a flat/shared house/student accommodation.*
- emotional resilience
  *Learning how to calm down when facing something unpleasant is crucial; how to deal with unexpected problems when living abroad; Skills help you to adapt quicker, adaptation skills, self-consciousness: to know yourself well is an asset if you are abroad.*
- open-mindedness to a new life environment
  *Country, people, university; willingness to challenge, motivation; socializing, problem solving skills; it is good to know about privileges, inequalities, racism, sexism and how to deal with (reverse) culture shock;*
- social skills:
  *If a person is more outgoing and open to new people, cultures and adventures, it will help you to have a nicer experience; dealing with different cultures and ways of living, being social, being open for getting to know new people; how to meet new people, ability to cooperate in a group*.
- intrapersonal skills:
  *Adaptability is essential; being organised, knowing how to deal with stress, having a proactive attitude, being capable of dealing with problems alone; confidence; you should be curious and eager to improve yourself to be able to get through a mobility period; time-management skills; coping with obligations, dealing with stressful situations, good organisational skills; basic problem-solving skills, planning and organisational skills.*

In interview, students expressed their opinions on the benefits of students’ international mobility. The answers allowed us to select the following categories of benefits:

- developing methods of learning
  *I can learn about different learning methods that are not available at my home university; Gain familiarity with a different teaching method; getting to know a different academic system; at my host university, they made us do presentations, speak and express our opinion first.*
- learning for career opportunities
  *I believe the most positive experience was attending a trade-fair, where I got to know and interact with others; I have experienced courses that have opened my eyes to new opportunities and new career perspectives.*
- intrapersonal learning
  *I become more independent; I developed self-confidence and independence; I was by myself in an unknown country all by myself, without any help; My most positive experience was going to a different city where I could communicate, solve my problems alone in a different language, overcoming challenges; I discovered that I can take care of myself while living in another country being outside of my comfort zone; I have more trust in myself now and that I can have a great life no matter where it will take me; more tolerant of differences and more curious.*

When asked to explain benefits of incorporating digital learning within mobility, students mentioned the digital learning environment and finding out what it is like to learn in a virtual environment. They saw limitations to digital learning such as the kinds of mobility that do not support social interactions, however, what is interesting is that the digital environment does not exclude social interactions. Some research participants saw opportunities in the digital environment like studying at a university of their own choice (called by one student “prestige university”), which will in their opinion, influence their career opportunities. They justified it as follows:

*Knowledge in the study field from digital tools does not offer social interactions and social life; I believe that physical mobility is the richest and digital learning would be lacking in most of the other aspects, such as making friends, the social and the learning side from that experience, and so knowledge would definitely be the most relevant; I would only take part if the course was conducted by a prestigious university;*

For some of our respondents, digital learning within mobility supports focus on knowledge in the study field, interesting programmes and courses, learning and career, and is perceived as a modern way of learning. A few examples from questionnaires, show how students describe their expectations of learning with digital tools during mobility:

*I will look into online mobility opportunities to gain valuable knowledge in my field; The programme content is the most important part to me if I do not live in another country; nowadays, most employers require knowledge of digital tools; Important for future career, to have better chances to obtain and develop global business employment contacts; digital mobility and digital learning can be helpful for my future; Learning and acquiring new skills are one of the biggest advantages of mobility as they offer an advantage at a career level in the future.*

In relation to international mobility learning, knowledge was referenced by students in terms of field of study, skills needed to use digital tools and communicate with others within the course, interpersonal attitudes (problem solving, communication with others) and intrapersonal attitudes (motivation for learning, being open to challenges in relation to a new learning environment).

Regarding learning, students mentioned new opportunities for skills development and information (knowledge) presentation. They associated online learning with personalized learning and the digital environment as a new tool for providing resources, and the way that knowledge can be efficiently presented and used for learning (permanent availability online):

*If I can’t understand something, I can watch again, listen to it again, but if I do not understand, I do not have anybody to ask; Digital environment as efficient learning environment, they saw new opportunities in digital learning; It is an extremely active learning opportunity; learning is quicker, very effective in terms of what you learn; Uploading of files, communicating on chat, and commenting verbally is best done in an online learning environment; It allowed better contact with professors, it was it quicker to sort out meeting online, connecting and asking.*

Adapting to a new digital environment is quick, however, this is often something that is undertaken intuitively by young people as evidenced by the terms now being used to describe them: “Digital Natives” (Prensky, 2001), “the Net Generation” or “Millennials”. Consequently, some commented that they didn’t feel that following a course outline in a digital environment was markedly different from traditional learning on university premises.

*I participate in face-to-face courses more often, but this was almost the same because work was organized, teachers organize live discussions during which I am always trying to raise my voice and voice my opinion; Moodle, blackboard, teachers provided pdfs, exercises, exams were written, but online, I feel that it was very similar to a physical course; The schedule was like physical learning.*

A recurrent topic that came up in focus group interviews was project group work, which due to its inherent qualities wherein social development is a way to get to know others was regarded as the optimal approach to working in a digital environment.

*Project work, it is better to work in group because I didn’t felt to be left alone; Good were meetings with colleagues on projects; In group projects, we have had tasks’ assignments, teachers told us deadline; Teachers were proactive and were facilitating group work; When working on the project, an online environment is better, as there are more documents distributed that we can study; What’s new is that the way I perceive personal relationships – meeting face-to-face changed and I don’t like to work that way anymore.*

International learning with digital tools is less related to working according to a planned schedule. Learners are more focused on achieving goals in their own way that supports independence in learning.

*It is completely different from ‘in person’ learning. It is worse, because the experience is different, even though professors make a big effort, it is still difficult to stay focused; I think digital learning requires a high level of self-motivation; When you learn online you need to motivate yourselves; I am more engaged in courses, and when my professors ask something, I always try to answer, I feel like I participate more in courses and tasks assigned.*

We asked students whether they would like to take part in digital learning again in future if the opportunity arises. 83 students (52%) out of 160 answered “Yes” to this question, 29 students (18%) chose “No” and 48 (30%) students marked “I don’t know”.

## Main findings – academic staff

Learning in the workplace allows employees to meet the challenges of the present day, such as rapid technological development, the globalization process and progressive socio-economic and cultural changes. It enables them to improve their qualifications and training after completing formal education. It can be done by participating in courses and training, as well as completely independently, e.g. by watching instructional films and reading books. Due to dynamic changes at universities, related mainly to their internationalization and new social expectations, becoming an entrepreneurial institution governed by the laws of academic capitalism (Clark, 1998), academic staff learn particularly intensively.

University employees prefer short-term mobility, mostly not longer than 2 weeks. 43% of the respondents indicated up to 2 weeks as being the most favourable. From 2 weeks to 2 months was in second place among the answers (34%), 2–6 months (11%), 6–12 months (6%), longer than 12 months (4%), different length (2%). HE staff would be the least willing to leave for longer than 12. The general conclusion is that short-term or medium-term international mobility is the most preferred and long-term mobility is the least. Such preferences can be explained by the fact that administrative staff and academic teachers do not want undertake intellectually engaging long-term commitments, which may result in a piling up of tasks and the need to work off the classes.

Just like in the case of students, we aimed at identifying the requirements that academic staff identify as necessary for international mobility. The general tendency is towards regarding digital and ICT knowledge and skills as the most needed. In terms of attitudes, the importance of having a positive attitude toward the learning process and digital tools and motivation to finish the mobility programme was emphasized by staff. In three areas: knowledge, skills and attitudes, we received the following responses:

- knowledge
  *Necessary digital knowledge or experience; Knowledge of the digital tools; Only basic ICT knowledge; IT knowledge; Computer knowledge and language skills (English); Self-organization ICT and language.*
- skills
  *Depends on the topic; The same as for other types of mobility; Digital skills; ICT and language skills; Basic ICT skills; Some ICT skills; A minimum level of digital skills is required to carry out this modality.*
- attitudes
  *A feeling that the course/learning is needed; Open mindedness; Willingness to learn, regularity, motivation for self-study; Motivation to finish the mobility programme; You should like digital tools; Patience and positive attitude to the learning process; Patience and focus.*

The most commonly indicated benefits were gaining digital skills (31%) and professional knowledge (29%). The average values were professional skills (9%), networking opportunities (7%), and others (8%). The remaining responses received less than 5% of responses.

Against the background of the above data and analyzes, the university employees' readiness to participate in mobility again is particularly surprising. Only 22% of respondents expressed their willingness to participate in digital mobility again, 37% were reluctant and 61% hesitant.

Assuming that the willingness to participate again is a measure of the attractiveness of learning, it can be concluded that for the respondents, digital learning was definitely the least attractive. This fairly sceptical attitude to digital learning may be explained by the time when the research was conducted (between October and December 2020). Empirical data were collected at the height of the COVID-19 pandemic while most academic staff were working online from home. It was a new situation for them. They were tired of social relations mediated by the digital tools, and expected direct interpersonal relations and traditional forms of personal development and learning.

## Analysis of students and staff perspectives

**Chart 6:**
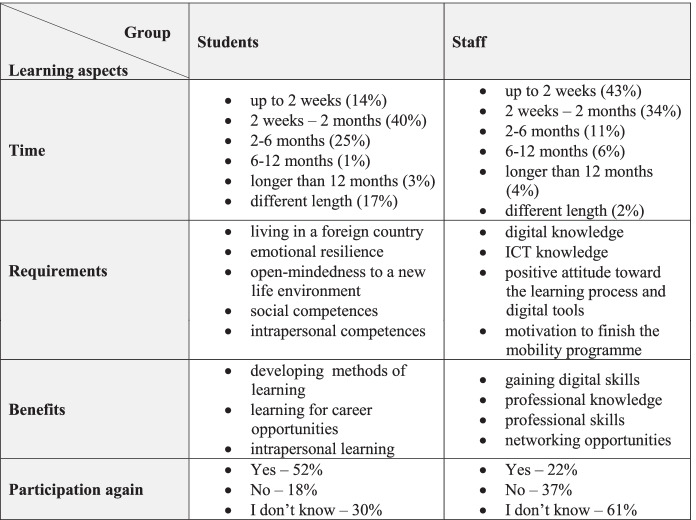
Learning within international mobility programmes – comparison perspectives of students and staff. **Source:** original study

Although students and staff represent one academic community, they differ slightly in their assessment of digital learning in international mobility (cf. Chart 6).

Regarding requirements for this type of learning, students pay more attention to “soft skills” (e.g. emotional resilience, open-mindedness), whereas staff express the opinion that “hard skills” like digital and ICT knowledge are of more value. There were also differences concerning benefits. Students benefit by taking advantage of intensive learning, whereas staff favour development through “hard skills”. The final difference resonates with the willingness to participate again: Both groups are similar in terms of preferred time, which is up to 2 weeks or 2 weeks – 2 months.

## Analysis of horizontal & vertical differentiation of learning background

**Chart 7:**
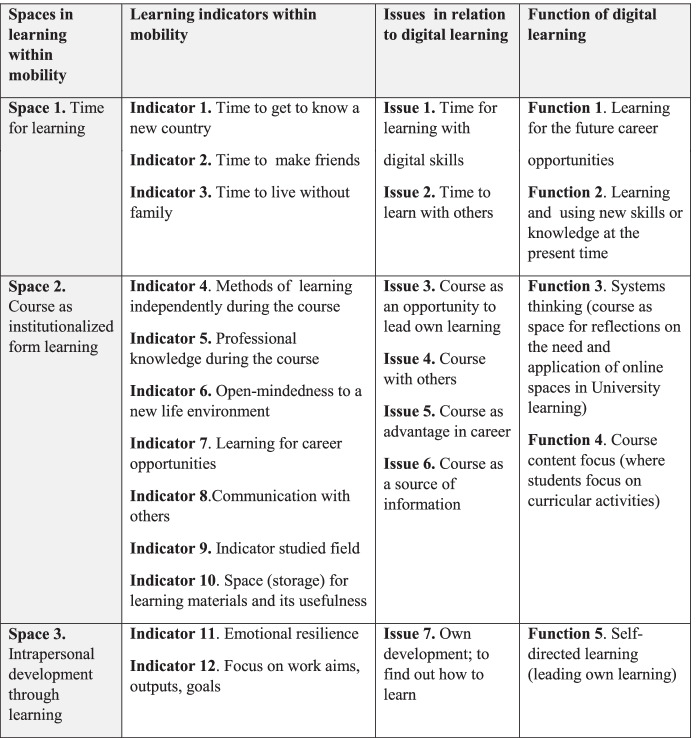
Analysis of mobility—traditional & digital mobility in relation to learning, horizontal & vertical differentiation of learning background. **Source:** original study

In our study, international mobility of students and staff was used to gather information on experiences with mobility in general (vertical differentiation of learning background – learning experiences gained at another university during mobility) and in focus group interviews – to gain information on learning in an digital environment (horizontal differentiation of learning background – digital tools as one of the opportunities used to gather learning experiences in academia). Comparison of learning within traditional mobility versus online learning in mobility enables us to identify the first distinctive spaces of both mobility schemes (indicators retrieved from content analysis of questionnaires and interviews), and then issues that relate to digital learning, which is what finally led to identifying the functions of digital learning (Chart 7).

## Results and discussion

Learning with digital technology is being described as a method to create a student-centered approach to learning (Tang & Chaw, 2016). Studies conducted in the academic community showed the influence of personalized learning with digital tools on academic goals (pursuing academic goals independently) and non-academic outcomes like interpersonal and intrapersonal development (Anthonysamy et al., 2020; Broadbent & Poon, 2015; Boelens, DeWever & Voet, 2017). Our study shows that the functions of international learning with digital tools are related to personalized learning. During interviews, students expressed their focusing on achieving goals in their own way that supports independence in learning, breaking away from a planned and time regimented schedule and management and resources. Students felt that being more open to learning opportunities strongly motivated them to learn and be open to discussion. They made a connection between personal changes and speaking skills, synthesis of information from online sources and perception of an online learning environment. Students were surprised at how easy learning in an international digital environment is, however, when telling us about the initial weeks, they complained about problems usually concerning the site of the university.

When considering participation in different mobility schemes, mobility with digital learning (digital courses) is being perceived as more advantageous for students or staff because of the economic factor of digital mobility: the economic side of mobility with no accommodation costs in the foreign country, and the career factor of digital mobility: obtaining a qualification that would make students stand out from other graduates in their chosen labour market. So the function of digital learning within mobility is to include wider groups of students and staff into international mobility exchange. This conclusion confirms previous findings that career identity is a compass for students’ and graduates’ actions to achieve professional goals (Fugate, Kinicki & Ashforth, 2004) and economic factors play an important role in students’ choices (Mazzarol and Soutar (2002).

In contrast to traditional learning, students within mobility that includes digital learning are more focused on knowledge and skills development in the study field, and their learning is visibly focused on intrapersonal development. Thus, the function of digital learning is to develop leadership in learning, with skills like problem solving, time management, independent project work and planning. Aspects relating to leadership in learning, a distinctive feature of digital learning within mobility are: (1) systems thinking, in which the experience of seeing how other universities have devised online systems/online courses/online working philosophy shapes how students understand those in their own universities, (2) self-directed learning, where the student as an individual takes independent and self-directed initiatives in identifying their needs (career opportunities), devising goals (to extend study knowledge), recognizing material (online resources), and human (the need to ask questions), (3) course content focus, where students focus more on curricular activities.

Weiss et al. (2006) consider learning with digital tools in terms of change. This change is perceived by comparing this environment with traditional learning. So, by comparing these two learning environments, Weiss et al. (2006) distinguish this learning environment by pointing out the change from traditional learning to learning with digital tools, the change from static learning to dynamic learning, from isolated learning to interactive learning, from private learning to public learning, from hidden learning to visible learning, and from exclusive learning to inclusive learning (Weiss et al. 2006). Our study expands these findings and shows changes in understanding how knowledge can be efficiently presented and used in a digital environment. In the opinion of students and academic employees, international learning in a digital environment changed their perception of opportunities that are offered by the mobility exchange programme. After experiencing international learning with digital tools, students and academic staff saw opportunities in knowledge development and course content that offer a digital environment (advantages of visual sources in digital learning *versus* written material within traditional mobility). Students appreciated the availability of materials, like presentations and noticed that they do not have to make notes during the course as all the materials (i.e. course scripts) were available online, so students could always have a look into the presentation for information. What is interesting, when considering international mobility exchange is that staff were more keen to share their knowledge and skills with others to support other mobility participants in their daily challenges at work.

Previous studies on students international mobility and learning (i.e. Clarke 2017; Fugate, Kinicki, Ashforth, 2004) emphasized that students became more resilient and more self-sufficient after participating in a mobility programme. Our study shows that functions of digital learning environments within a mobility programme are related to the development of students’ motivation to learn, and independence in gaining knowledge in their chosen field. In international digital learning, the online courses of which are characterized by their innovative pedagogical and assessment practices, students are required to be able to adapt, and more specifically to adapt to a challenging academic environment. In other words, a challenging learning environment helps students become more autonomous in their learning, and more willing to open up to meeting the challenges encountered in various educational settings.

Although university staff and students belong to one academic community, they differ little in their assessment of digital learning in international mobility. Students, pay more attention to “soft skills”, whereas for staff, “hard skills” available for immediate use at work are more important. Both groups pay more attention to administrative problems and to the challenges students face when learning digitally in cyberspace. The purpose of learning within a mobility programme is to develop skills beyond the programme of study to solve these problems. These skills can be used not to pass the exams but in the future in the labour market, which is increasingly moving into cyberspace. This is a specific “added value” of students’ digital learning. Members of the academic community have the need to express themselves during online courses, which as they explained, was not often possible within the courses they experienced during their past mobility that had included traditional learning without digital tools. For them, digital learning means translating new context (intranationalization; translation by applying information received from course content/document) to their own activity and extension of the course content (externalization) meaning such functions such as oral expression, like discussing, interacting with teachers and peers.

## Availability of Supplementary Materials

University of Lodz Repository.

## Data Availability

Metadata are available at the Repository of University of Lodz.
